# Effective Teaching Behaviors of Clinical Nursing Teachers: A Qualitative Meta-Synthesis

**DOI:** 10.3389/fpubh.2022.883204

**Published:** 2022-04-28

**Authors:** Jian Zhang, Fenhua Zhou, Jinxia Jiang, Xia Duan, Xin Yang

**Affiliations:** ^1^Health School (Jinshan), Shanghai University of Medicine and Health Sciences, Shanghai, China; ^2^Emergency Department, Shanghai Tenth People's Hospital, Tongji University School of Medicine, Shanghai, China; ^3^Nursing Department, Shanghai First Maternity and Infant Hospital, Tongji University School of Medicine, Shanghai, China; ^4^Department of Traditional Chinese Medicine, Shanghai Tenth People's Hospital, Tongji University School of Medicine, Shanghai, China

**Keywords:** clinical nursing teachers, effective teaching behaviors, qualitative research, meta-synthesis, nursing students

## Abstract

**Objectives:**

To identify, appraise, and synthesize the available evidence exploring the effective teaching behaviors of clinical nursing teachers.

**Design:**

The Joanna Briggs Institute (JBI) guidelines were followed, and a meta-synthesis was conducted.

**Review Methods:**

Following databases were searched for relevant qualitative studies published in English and reporting primary data analysis, including experiences and perceptions of nursing students: PubMed, EBSCOhost, OVID, etc. Qualitative Assessment and Review Instrument were used to pool the qualitative research findings. Through the repeated reading of the original literature, the similar findings were combined and sorted into new categories, and then summarized into different synthesized themes.

**Results:**

A total of nine articles were included. The review process produced 29 subcategories that were aggregated into seven categories. The categories generated three synthesized findings: good teaching literacy, solid professional competence, and harmonious faculty-student relationship.

**Conclusions:**

The effective teaching behaviors of clinical nursing teachers are the driving force for the progress and growth of nursing students. In order to improve the effectiveness of clinical nursing teaching, nursing teachers should be fully aware of effective teaching behaviors for nursing students to master nursing theories and skills.

## Introduction

Nursing education includes both theoretical and practical educational aspects. Nursing students learn how to provide care in different settings, such as classrooms and clinic (1). Nursing education has a strong practicality, as it allows nursing students to better master theoretical knowledge, accumulate practical experience, and finally develop from nursing students to qualified professional nurses through clinical practice.

Clinical practice is an important stage for nursing students to complete the transformation of nurses' role in psychology and behaviors. As an important part of nursing education, high-quality teaching of clinical practice is very important to cultivate qualified clinical nursing students. A previous study has shown that communication ability, health education ability, and professional psychological quality are relatively strong among nursing students, while their clinical scientific research, clinical management, and clinical teaching are relatively weak (2). In addition, according to the results of an interview with students on the soft environment evaluation of clinical nursing teaching, the teaching evaluation system of clinical nursing teachers needs to be further updated and improved; the working environment is harmonious, but opportunities for nursing students to participate in clinical practice are insufficient; teachers have a strong professional ability, but the teaching level needs to be further improved; teaching contents and methods are limited, and the learning effect is not ideal (3). Consequently, it is of urgent importance to improve the quality of clinical teaching.

The quality of clinical teaching does affect not only the future value orientation of nursing students but also the cultivation of their professional quality and career planning (4). In clinical teaching of nursing practice, nursing teachers have a leading role, and their teaching behaviors directly affect the quality of clinical teaching. In order to help nursing students become excellent nurses, clinical nursing teachers must use educational theories that are more in line with teachings from clinical practice to improve teaching methods, such as humanistic theory (5). Education should completely focus on nursing students and include teaching and learning activities that are consistent with the learning needs of nursing students so as to effectively improve the quality of clinical teaching.

Nursing students are the main focus of teaching activities, and nursing students tend to make different judgments on the effectiveness of clinical nursing teachers' behaviors based on their different learning motivations, learning strategies, cognitive styles, and family background (6). A previous study has shown that in the process of teaching, a full understanding of nursing students' learning characteristics by nursing teachers, cognitive style, and teaching effectiveness evaluation can effectively guide nursing teachers to adjust teaching methods and to teach activities to meet the teaching needs of different groups (7). Therefore, clinical nursing teachers should improve clinical teaching behaviors considering nursing students' perspective to cultivate high-quality nursing talents.

Teachers used theoretical and practical approaches in the process of teaching, which combined with their own qualities and teaching methods adopted in order to promote nursing students' learning and achieve their predetermined learning goals are considered to make up effective teaching behavior (8). The effective teaching behaviors of clinical nursing teachers can improve the clinical ability of nursing students and improve the effectiveness of clinical practice, thus ensuring the teaching quality (9). Therefore, in order to further improve the quality of clinical nursing teaching, it is very important to acquire an in-depth understanding of the effective teaching behaviors of clinical nursing teachers. The purpose of this study was to integrate the existing qualitative research results with meta-synthesis data in order to provide a reference for improving the quality of clinical nursing teaching.

## The Review

### Objective

The objective of this exploratory qualitative meta-synthesis was to determine the experience of nursing students and their perception of effective teaching behaviors of clinical nursing teachers. This review enabled us to make recommendations to further improve the effectiveness of clinical nursing teaching.

### Design and Search Strategy

A systematic literature search according to the Joanna Briggs Institute (JBI) Reviewers' Manual (10) and a priori protocol (11) was performed. We initiated a three-step search strategy and followed a focused question. To guide the structure and identify the key aspects of the search, we adopted mnemonic for qualitative reviews. The target phenomenon was an investigation of nursing students' experiences and perceptions of effective teaching behaviors of clinical nursing teachers in their clinical practice, which has inspired the research question, the definition of the Population, the Phenomenon of Interest, the Context and the Type of study (PICoS) of the review. We searched PubMed, EBSCOhost, OVID, Embase, Scopus, Web of Science and ProQuest for qualitative studies on the effective teaching behaviors of clinical nursing teachers from the establishment of these databases to February 2022. CareSearch and Google Scholar were also utilized for searching the gray literature. The following keywords were included: clinical nursing, effective teaching, effective teaching behaviors, nursing students, qualitative research, qualitative study, interview, feelings, experience and perception. The systematic questions and search terms based on our PICoS are shown in Table 1. The qualitative research assessment and evaluation tool JBI-QARI online application software was used to evaluate the literature quality, extract the research results and comprehensive research results. This study met the requirements of the Helsinki Declaration.

**Table 1:** PICoS.

**Types of participants (*P*)**	**Types of phenomena of interest (*I*)**	**Types of contexts (Co)**	**Types of studies (*S*)**
This review will investigate nursing students.	This review will investigate nursing students' experiences and perceptions on effective teaching behaviors of clinical nursing teachers in their clinical practice.	This review will investigate nursing education in the practical process.	This review will focus on qualitative studies.
Systematic search terms are “nursing students, nurse students, student nurses, pupil nurses.”	Systematic search terms are “effective teaching behaviors, effective teaching strategies, effective teaching, teaching effectiveness, effective teaching methods, nursing faculty, nurse faculty, nursing educator, nurse educator.”	Systematic search terms are “clinical nursing, nursing education.”	Systematic search terms are “qualitative research, qualitative study, interview, feelings, experience, perception.”

### Critical Appraisal

Before being included in the review, the two first authors independently assessed the validity of the literature using the Joanna Briggs Institute Qualitative Assessment and Review Instrument (JBI-QARI) (12, 13). The disagreements that arose between the two authors were resolved through discussion or by the third author.

### Data Extraction and Synthesis

The data that were extracted from the papers using the JBI-QARI data extraction tool included specific details about nursing students' experiences and perceptions on effective teaching behaviors of clinical nursing teachers in their clinical practice. The researchers individually reviewed the nine studies and extracted the necessary information by using the JBI procedure for meta-synthesis (10). The similar findings were combined and sorted into new categories, and then summarized into different synthesized themes (12).

## Results

**Figure 1:**
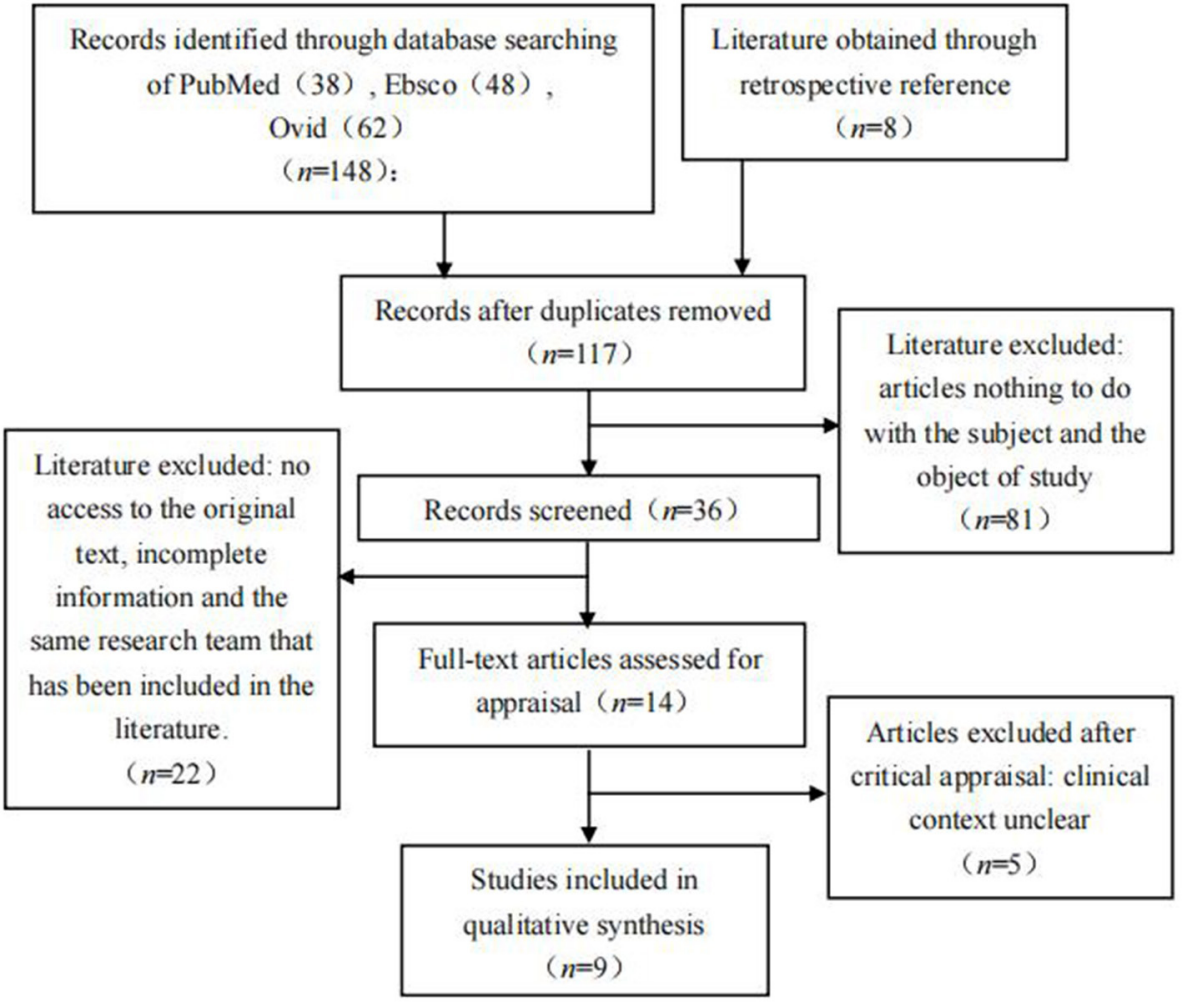
PRISMA flowchart.

A total of 156 papers were retrieved from databases. Hundred and seventeen papers were screened for relevance based on subject and object after duplicates were removed, and 36 full-text papers were screened for eligibility. Twenty-two papers were excluded for the following reasons: no access to the original text, incomplete information, and the same research team that has been included in the literature. Among 14 papers that were assessed for appraisal, five were excluded. Of these, nine papers were included in the qualitative meta-synthesis. Figure 1 shows the PRISMA flow diagram of the process of retrieval and selection of papers for inclusion (13).

The nine included studies used qualitative methodologies. They were conducted between 2006 and 2019 in eight different countries, and all were published in English. An illustration of the participants, designs, aims, methods of analysis, and key findings is presented in a meta-summary (Table 2).

**Table 2:** Meta-summary of included studies.

**Author (year) Country**	**Aim**	**Participants and design**	**Methods/analysis**	**Key findings**
Pearson et al. (14) (2011) UK	To explore the perceptions of clinicians, clinical learners, and practice staff of key elements of being a teaching practice.	28 clinical learners, including postgraduate nurses and others Phenomenology	Individual face-to-face interviews or focus group interviews Inductive analysis	Two themes emerged: a positive learning environment (support for learning, excellence in teaching); learning culture (a passion for education).
Jiang et al. (15) (2018) PRC	To explore effective teaching methods in the emergency department from the perspective of Millennial nursing students in Shanghai.	16 nursing students from six colleges of nursing and five nursing high schools in Shanghai Qualitative study	Semi-structured interviews Colaizzi's seven-step data analysis	Three themes emerged: demonstrating harmonious faculty-student relationship, possessing professional competence, and being empathetic for teaching.
Lovrić et al. (16) (2017) Croatia	To explore what competencies BSc nursing students expect from their clinical faculties and whether their expectations changed.	34 BSc nursing students A two-phase, mixed-methods design	Reflections on the expectations Inductive analysis	Four themes emerged: a higher level of teaching ability; positive human qualities; clinical faculties' professional evaluation of the student; good interpersonal relations.
Harms et al. (17) (2019) Canada	To fill this gap by examining narrative comments from psychiatry faculty evaluations to understand learners' perceptions of educator effectiveness.	324 undergraduate and postgraduate learners from McMaster University A fundamental qualitative descriptive design	Narrative evaluation Inductive analysis	Four themes emerged: personal characteristics (learner-centered, supportive, engaging, good communicator, respectful, professional); relationships matter (learner security-the conditions for optimal learning, a spectrum of admiration); person as pedagogy (medical teachers themselves being the method of teaching); supervisors-more than medical experts (skills and qualities building upon their knowledge base).
Kelly (18) (2007) Canada	To elicit learner's views of what teacher characteristics and contextual influences impact them in clinical settings.	30 students at the end of second and third years Qualitative study	In-depth interviews Phenomenographic analysis	Three themes emerged: clinical teacher knowledge; feedback and communication skills (teacher's listening skills, a respectful, calm, co-learner, being straightforward and honest); environmental factors (ideal student-teacher ratios, welcoming students and trying to help them out, the importance of peer support).
Gustafsson et al. (19) (2015) Sweden	To describe and compare the clinical teacher's role in different models of clinical practice from the perspective of nursing students.	8 nursing students in the qualitative part of the study A mixed-method study A quantitative study with comparative design and a qualitative study with descriptive design	A mixed-method Inductive analysis	Three themes emerged: enabling integration of theory and practice; co-operation between placement staff and nurse teacher (being like a member of the nursing team, transmitting his or her pedagogical expertise to the clinical team); the relationship between student, mentor, and nurse teacher (The common meetings between myself, mentor and NT being comfortable experience, a climate of the meetings being congenial, focus on the meetings being in my learning needs).
McSharry et al. (20) (2017) Ireland	To explore the clinical teaching and learning within a preceptorship model in an acute care hospital in Ireland and identify when best practice principles occurred.	13 student nurses from 1st, 3rd, and 4th year from each of the four clinical sites A qualitative research study	semi-structured interviews Inductive analysis	Five themes emerged: continuity-foundation for effective teaching and learning relationship (within a relationship of mutual interest and respect); talking through practice; assessing practice-scaffolding learning (exploratory conversations); continuous assessment of the students understanding and performance; teaching clinical reasoning-preceptors' questions (the usefulness of critical questioning in developing student nurses' clinical reasoning skills in the context of clinical practice).
Yousefy et al. (21) (2015) Iran	To explore the environment of clinical baccalaureate nursing students' education.	54 nursing students and eight clinical educators from the four geographically diverse universities A qualitative study	Individual interviews, focus groups, and direct observations A content analysis	Two themes emerged: questions not being challenging and incentive to improve critical thinking in students; incompetency of clinical educators (not prepared and competent for being a role model practical setting).
Günay et al. (22) (2018) Turkey	To determine the transfer of theoretical knowledge into clinical practice by nursing students and the difficulties they experience during this process.	30 nursing students in a university located in the east of Turkey A qualitative research	Focus group interviews The method of content analysis	Three themes emerged: guidance and communication (inadequacy in receiving clinical guidance, lack of appreciation, cooperation); clinical evaluation (expectations changing based on the instructor, injustice in clinical grading); expectations (to be active in clinical education, love their profession and feel appreciated, to accompany them in the clinical area).

### Meta-Synthesis

**Figure 2:**
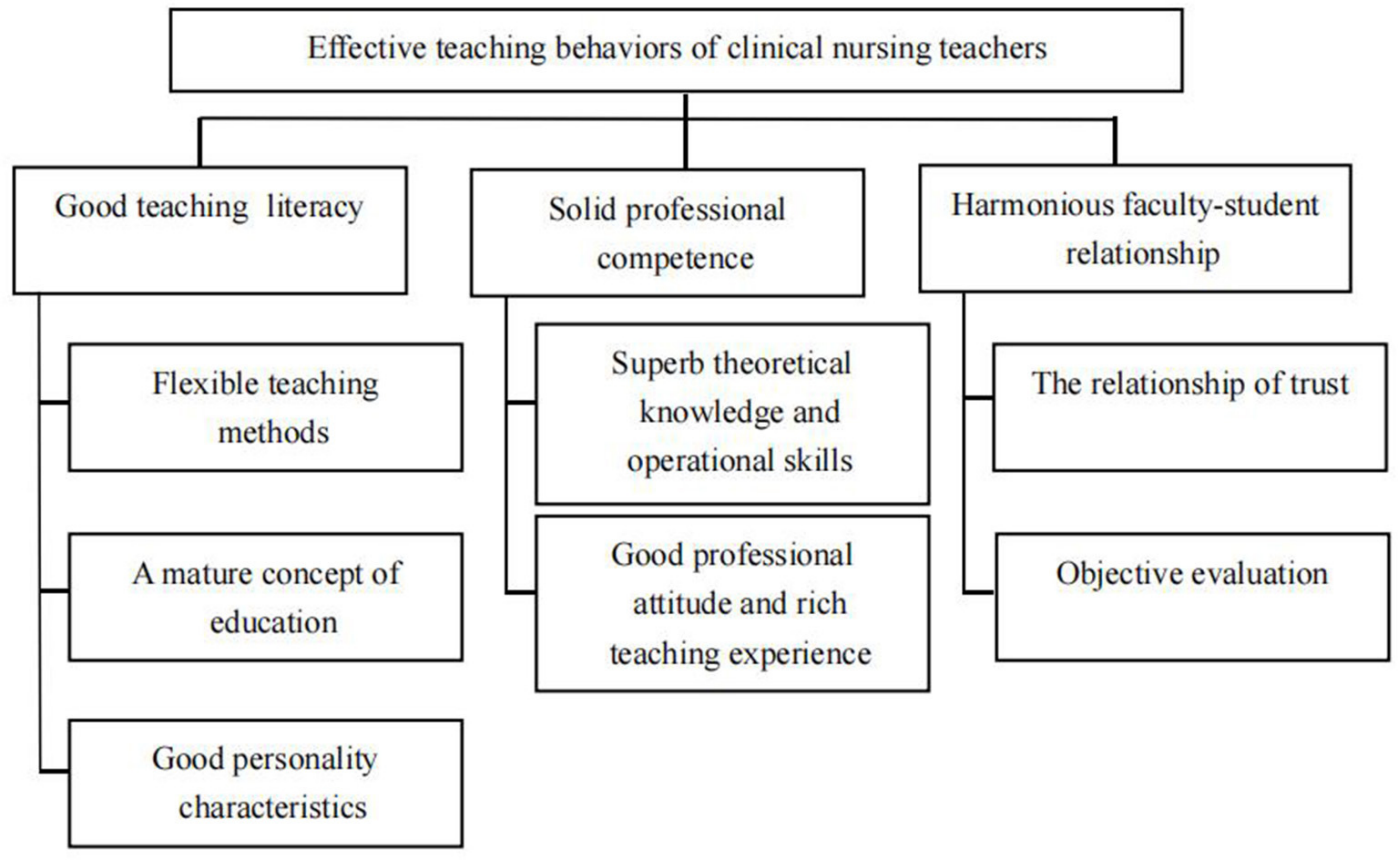
The relation between meta-synthesis, themes, and categories in the review.

**Figure 3:**
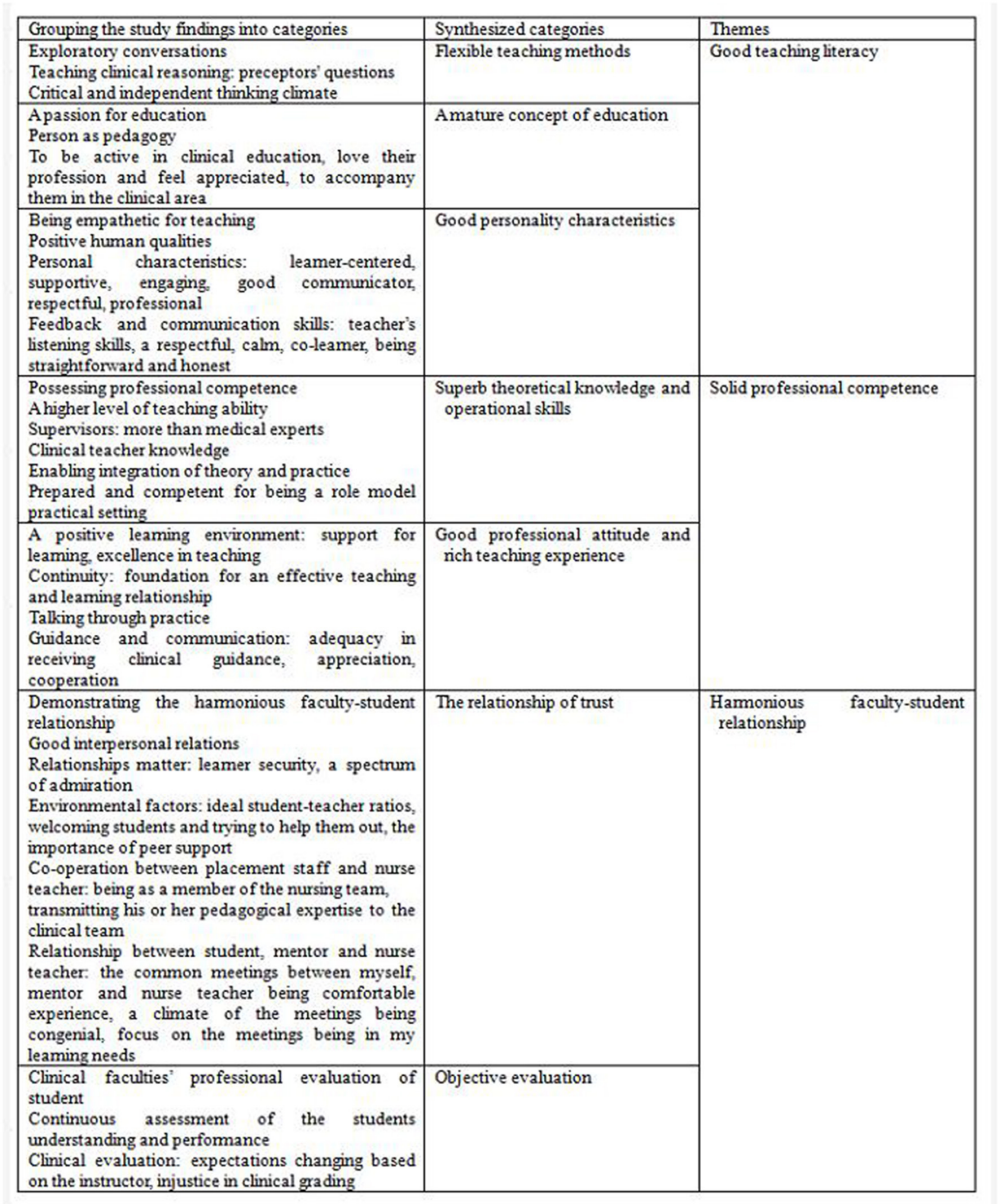
The meta-aggregative approach from grouping the study findings into categories and synthesizing the categories into themes.

This systematic review presented the following meta-synthesis: nursing students' experiences of effective teaching behaviors of clinical nursing teachers in their clinical practice. The meta-synthesis had three themes: (1) good teaching literature, (2) solid professional competence, and (3) harmonious faculty-student relationship. The three themes had seven synthesized categories (Figure 2) that were derived from 29 grouped study findings. The grouping of the findings into synthesized categories is illustrated in Figure 3. One of the three themes had a frequency effect size of 100% (23), while other themes had effect sizes of 89 and 78% (Table 3).

**Table 3:** The effect size of themes.

**References**	**Themes**		
	**Good teaching literacy**	**Solid professional competence**	**Harmonious faculty-student relationship**
Pearson et al. (14)	x	x	
Jiang et al. (15)	x	x	x
Lovrić et al. (16)	x	x	x
Harms et al. (17)	x	x	x
Kelly (18)	x	x	x
Gustafsson et al. (19)		x	x
McSharry et al. (20)	x	x	x
Yousefy et al. (21)	x	x	
Günay et al. (22)	x	x	x
%	89	100	78

### Good Teaching Literacy

Following themes were derived from 10 grouped study findings and three synthesized categories: flexible teaching methods, a mature concept of education, and good personality characteristics.

#### Category 1: Flexible Teaching Methods

Most clinical nursing teachers are well prepared for teaching. They can show clear teaching objectives and learning priorities and can apply flexible teaching methods to create a free learning environment. According to one student: “Good teachers make you do things. They're the ones that would assign you a patient and tell you what to do. They will be watching you if you have any problems.” (20) (p78).

Teachers should take advantage of the opportunity of close contact with nursing students to stimulate their learning enthusiasm so as to fully tap into their full potential and realize the multi-dimensional interaction between teachers and students. Another student said: “We believe that this kind of questioning (such as how, why or what if) helped students to verbalize, and hence refine their knowledge.” (20) (p79).

#### Category 2: A Mature Concept of Education

Clinical nursing teachers need to establish the educational concept of “humanism”. Their teaching literature is the foundation of vocational teaching and the key supporting point of teaching activities, which has a significant impact on the teaching effect and the development of nursing students. The following quote described this reflective process, “One of the most engaging seminars I have participated in via video conference. This speaks highly of the teacher's ability to facilitate thoughtful discussion, present material, and also be conscious of how this transmits over technology. Superb!” (17) (p23).

At the same time, teachers need to be sympathetic, and nursing students often like teachers who show sustained enthusiasm for teaching. A student said, “They like to teach and they are willing to teach anybody who wants to listen…” (14) (p162).

#### Category 3: Good Personality Characteristics

The overall personality characteristics of clinical nursing teachers are good. They are showing enthusiasm, optimism, neatness, and modesty. They are active and creative thinkers, able to control their emotions, and having a strong ability to cope with setbacks and failures: a female student stated, “Deep down, I just want to be as a good nurse as she is…so gentle…and bringing warmth to the hopeless…” (15) (p223).

In short, teachers' charisma can improve the learning experience of nursing students. A student recalled, “The educator really soothed our anxiety levels…” (17) (p21).

### Solid Professional Competence

The following themes were derived from 10 grouped study findings and two synthesized categories: superb theoretical knowledge and operational skills, and good professional attitude and rich teaching experience.

#### Category 4: Superb Theoretical Knowledge and Operational Skills

The good professional attitude and skills of clinical nursing teachers are the catalyst and activator of teaching effect that further the development of students' ability. One student commented: “The thing that impressed me the most was her knowledge level and the way she incorporated it - put it into practice. She's got to be able to pull the whole thing together.” (18) (p889).

The clinical skills, professional knowledge, professional responsibility and the attitude shown by clinical teachers set an example for nursing students. A student concurred, “You have to be ready to handle different unpredictable emergency situations in the ED. Sometimes you need to act like a senior supervisor…” (15) (p223).

#### Category 5: Good Professional Attitude and Rich Teaching Experience

Nursing students expect clinical nursing teachers to act as their role models and to influence nursing students through their actions. A fourth-year student said, “Everyone is really friendly, and you share a staff room with them and stuff…they kind of chat with you as you were another member of staff really…” (14) (p161).

However, individual teachers also reprimand nursing students, which may increase the psychological burden in nursing students. A student stated, “If they appreciated us, we would like our profession and would be highly motivated.” (22) (p83). This suggests that teachers should give more care to nursing students.

### Harmonious Faculty-Student Relationship

The following themes were derived from nine grouped study findings and two synthesized categories: the relationship of trust and objective evaluation.

#### Category 6: The Relationship of Trust

Nursing students expect clinical nursing teachers to have an amiable attitude and give them full encouragement and respect. A student recalled the situation, “One day, I encountered my teacher from the ICU in the cafeteria of the hospital. I felt so excited and was deeply moved when she called my name…” (15) (p222).

This harmonious teacher-student relationship helps to provide nursing students with a good learning atmosphere, not only to practice and develop their clinical skills independently but also to get a sense of security from the stable relationship between teachers and students. A learner commented, “It fosters personal growth and motivates the learner to do better.” (17) (p21).

#### Category 7: Objective Evaluation

Clinical nursing teachers are busy and cannot timely and effectively make an objective evaluation of nursing students, which has a role in motivating nursing students. Some students complained, “Some assistants try to look for our mistakes instead of helping us overcome our gaps in knowledge.” (22) (p84).

Evaluation behaviors on behalf of the teachers is of vital importance, and can impact the effectiveness of clinical learning of nursing students. Therefore, clinical nursing teachers need to have rich teaching skills, which is of great significance for the motivation and growth of nursing students. A student recalled, “…My preceptor asked me ‘so a patient is being discharged, what do you do?' I was so nervous…But then I answered and she was like ‘well done, you've got the necessary knowledge. Now go and do it.” (20) (p78).

## Discussion

The results of meta-syntheses, which are considered scientific and reliable, can complement qualitative research results (24). Compared with quantitative research, qualitative research can reflect more humanistic care, and it is becoming increasingly used in health care, health education, health service, and nursing (25). Herein, we conducted a systematic review of the qualitative research on the effective teaching behaviors of clinical nursing teachers. Through critical appraisal, the qualitative research results from nine articles were identified, summarized, and integrated by meta-synthesis, and the essence of the phenomenon was deeply explored. Seven categories were formed and synthesized into three themes so as to fully understand nursing students' experiences of effective teaching behaviors on behalf of clinical nursing teachers and find a comprehensive explanation for the consistencies between the results of various qualitative studies. The meta-synthesis process of this study was rigorous, and the results are reliable, which can be helpful for improving the quality of clinical nursing teaching and can be used as the application basis for evidence-based practice.

Cultivating good teaching literacy and strengthening teaching supervision is of essential importance. Clinical nursing teachers should establish their own role, not only as role models for clinical nurses, but also as knowledgeable and reliable teachers (26). The research shows that finding a role model is an important characteristic of effective teaching behaviors; thus, clinical teachers should nurture the characteristics of self-discipline, tolerance, self-improvement, politeness, and similar (27). Clinical nursing teachers should strive to create a good learning environment for nursing students, and their flexibility and openness might benefit nursing students a lot. Clinical teaching hospitals should strengthen cooperation with medical colleges and universities, make full use of their teaching advantages and carry out relevant training to help teachers improve their teaching abilities of clinical nursing. Teaching management departments should encourage clinical nursing teachers to participate in higher-level academic education and take courses such as psychology and pedagogy, which can help them master flexible teaching methods, own mature concepts of education, and enhance their awareness of professional development. The teaching management department should identify the necessary teaching qualifications of clinical nursing teachers and adopt the appointment system to strengthen teachers' responsibilities and abilities and ensure good teaching quality. The teaching management department can select students in accordance with their aptitude and strengthen the supervision of teaching quality so as to help nursing students make further achievements.

Paying attention to solid professional competence and advocating personalized teaching is of vital importance. In clinical nursing teaching, nursing students are the main focus of learning activities, and clinical nursing teachers are guides (28). The effective teaching behaviors of teachers are very important for the learning and growth of nursing students, which directly affects the effectiveness of teaching (29). Therefore, clinical nursing teachers should have superb theoretical knowledge and operational skills. At the same time, clinical nursing teachers should strengthen their professional attitude, have a positive attitude toward their clinical nursing teaching, and set an example for nursing students through words and deeds. Under the correct guidance of teachers with solid professional skills, nursing students can feel grounded and safe. Moreover, nursing students are more likely to admire the various professional skills of their teachers and their ability to communicate, thus being encouraged to study actively. The research shows that most nursing students recognize the theoretical knowledge and operational skills of clinical nursing teachers, but clinical teachers often ignore the differences between theoretical and practical courses learned by trainee nursing students from different schools, levels, and regions (30). Therefore, when arranging nursing teachers in clinical nursing teaching, the teaching management departments should fully consider the knowledge needs of nursing students with different backgrounds in clinical learning and pay attention to personalized and hierarchical teaching.

Establishing a harmonious faculty-student relationship and allocating teachers rationally are also essential factors. In addition to good teaching literature and solid professional competence, strong communication between teachers and students is very important to the clinical practice of nursing students (31). Some studies have shown that nursing students are faced with problems such as unfamiliar hospital environments, tense interpersonal relationships, and low self-identity in clinical practice (32). These problems could be solved by the encouragement of clinical nursing teachers in all aspects. Also, it is particularly important to establish a harmonious relationship between teachers and students. As clinical nursing teachers are most concerned about what nursing students have really learned, while nursing students pay special attention to whether teachers can evaluate them fairly (33), clinical nursing teachers should have the skills to evaluate nursing students and be able to guide them at any time. However, besides clinical teaching, clinical nursing teachers also undertake heavy clinical work. Therefore, many factors can cause great pressure on clinical nursing teachers, and the emergence of work fatigue can seriously affect their enthusiasm for nursing work. This requires teaching hospitals to establish a standardized evaluation mechanism for clinical teaching teachers and to rationalize the allocation of nursing human resources to prevent clinical nursing teachers from professional exhaustion and burnout. At the same time, teachers should also strive to maintain a good leading spirit in clinical nursing teaching.

Dewey put forward the teaching theory of “learning by doing,” which requires nursing teachers and nursing students to build a harmonious faculty-student relationship. Teachers are the guides and leaders of nursing students' learning, rather than simple knowledge instillers and transmitters. As the instructor of nursing students, teachers are required to have good teaching literacy and solid professional competence in order to guide the study and life of nursing students and promote their all-round physical and mental development.

## Study Limitations

The limitations of this review are related to the number of included literature and search strategies. Although the search strategy is extensive, some related literature may have been left out.

## Conclusion

To sum up, compared with quantitative research, qualitative research can reflect more subjective aspects (34). The effective teaching behaviors of clinical nursing teachers greatly affect the nursing students, representing the driving force for nursing students' progress and growth. Clinical nursing teachers and teaching administrators should be fully aware of the value of effective teaching behaviors in guiding nursing students to master nursing theories and skills so as to improve the effectiveness of clinical nursing teaching further.

## Data Availability Statement

The original contributions presented in the study are included in the article/Supplementary Material, further inquiries can be directed to the corresponding author/s.

## Author Contributions

JJ and JZ: study design. JZ, FZ, JJ, XD, and XY: data collection, data analysis, and manuscript preparation. All authors contributed to the article and approved the submitted version.

## Funding

This work was supported by the Medical Educational Reform Project of Tongji University (Grant Reference No. 2021YXSZ01). The funding source paid for all costs associated with the development and the publishing of the present manuscript.

## Conflict of Interest

The authors declare that the research was conducted in the absence of any commercial or financial relationships that could be construed as a potential conflict of interest.

## Publisher's Note

All claims expressed in this article are solely those of the authors and do not necessarily represent those of their affiliated organizations, or those of the publisher, the editors and the reviewers. Any product that may be evaluated in this article, or claim that may be made by its manufacturer, is not guaranteed or endorsed by the publisher.
