# Supplementary material for: Effective Teaching Behaviors of Clinical Nursing Teachers: A Qualitative Meta-Synthesis
Source: Front Public Health. 2022 Apr 28;10:883204. doi: 10.3389/fpubh.2022.883204 (PMC9095952; doi:10.3389/fpubh.2022.883204)
Supplement: Supplementary file 1 [file Table_1.pdf]

**Table 1. PICoS**

| Types of participants (P) | Types of phenomena of interest (I) | Types of contexts (Co) | Types of studies (S) |
|---|---|---|---|
| This review will investigate nursing students. | This review will investigate nursing students' experiences and perceptions on effective teaching behaviors of clinical nursing teachers in their clinical practice. | This review will investigate nursing education in the practical process. | This review will focus on qualitative studies. |
| Systematic search terms are "nursing students, nurse students, student nurses, pupil nurses". | Systematic search terms are "effective teaching behaviors, effective teaching strategies, effective teaching, teaching effectiveness, effective teaching methods, nursing faculty, nurse faculty, nursing educator, nurse educator". | Systematic search terms are "clinical nursing, nursing education". | Systematic search terms are "qualitative research, qualitative study, interview, feelings, experience, perception". |

**Table 2.** Meta-summary of included studies

| Author（year）<br>Country | Aim | Participants and design | Methods/analysis | Key findings |
|---|---|---|---|---|
| Pearson et al（14）(2011)<br>UK | To explore the perceptions of clinicians, clinical learners, and practice staff of key elements of being a teaching practice. | 28 clinical learners, including postgraduate nurses and others<br>Phenomenology | Individual face-to-face interviews or focus group interviews<br>Inductive analysis | Two themes emerged: a positive learning environment （ support for learning, excellence in teaching ）; learning culture （a passion for education）. |
| Jiang et al（15）(2018)<br>PRC | To explore effective teaching methods in the emergency department from the perspective of Millennial nursing students in Shanghai. | 16 nursing students from six colleges of nursing and five nursing high schools in Shanghai<br>Qualitative study | Semi-structured interviews<br>Colaizzi's seven-step data analysis | Three themes emerged: demonstrating harmonious faculty-student relationship, possessing professional competence, and being empathetic for teaching. |
| Lovrić et al（16）(2017)<br>Croatia | To explore what competencies BSc nursing students expect from their clinical faculties and whether their expectations | 34 BSc nursing students<br>A two-phase, mixed-methods design | Reflections on the expectations<br>Inductive analysis | Four themes emerged: a higher level of teaching ability; positive human qualities; clinical faculties' professional evaluation of the student; good interpersonal relations. |

| | | | | |
|---|---|---|---|---|
| | changed. | | | |
| Harms et al（17）(2019)<br>Canada | To fill this gap by examining narrative comments from psychiatry faculty evaluations to understand learners' perceptions of educator effectiveness. | 324 undergraduate and<br>postgraduate learners from McMaster University<br>A fundamental qualitative descriptive design | Narrative evaluation<br>Inductive analysis | Four themes emerged: personal characteristics （ learner-centered, supportive, engaging, good communicator, respectful, professional ）; relationships matter（learner security-the conditions for optimal learning, a spectrum of admiration); person as pedagogy（medical teachers themselves being the method of teaching）; supervisors-more than medical experts（skills and qualities building upon their knowledge base）. |
| Kelly（18）(2007)<br>Canada | To elicit learner's views of what teacher characteristics and contextual influences impact them in clinical settings. | 30 students at the end of second and third years<br>Qualitative study | In-depth interviews<br>Phenomenographic analysis | Three themes emerged: clinical teacher knowledge; feedback and communication skills (teacher's listening skills, a respectful, calm, co-learner, being straightforward and honest ）; environmental factors （ ideal student-teacher ratios, welcoming students and trying to help them out, the importance of peer support）. |
| Gustafsson et al（19）(2015)<br>Sweden | To describe and compare the clinical teacher's role in different models of clinical practice from the perspective of nursing students. | 8 nursing students in the qualitative part of the study<br>A mixed-method study<br>A quantitative study with comparative design and a qualitative study with descriptive design | A mixed-method<br>Inductive analysis | Three themes emerged: enabling integration of theory and practice; co-operation between placement staff and nurse teacher（being like a member of the nursing team, transmitting his or her pedagogical expertise to the clinical team); the relationship between student, mentor, and nurse teacher（The common meetings between myself, mentor and NT being comfortable experience, a climate of the meetings being congenial, focus on the meetings being in my learning needs）. |
| McSharry et al（20）(2017)<br>Ireland | To explore the clinical teaching and learning within a preceptorship model in an acute care hospital in Ireland and identify when best practice principles occurred. | 13 student nurses from 1st, 3rd, and 4th year from each of the four clinical sites<br>A qualitative research study | semi-structured interviews<br>Inductive analysis | Five themes emerged: continuity-foundation for effective teaching and learning relationship（within a relationship of mutual interest and respect); talking through practice; assessing practice-scaffolding learning （exploratory conversations）; continuous assessment of the students understanding and performance; teaching clinical reasoning-preceptors' questions （ the |

| | | | | |
|---|---|---|---|---|
| | | | | usefulness of critical questioning in developing student nurses' clinical reasoning skills in the context of clinical practice）. |
| Yousefy et al（21）(2015)<br>Iran | To explore the environment of clinical baccalaureate nursing students' education. | 54 nursing students and 8 clinical educators from the four geographically diverse universities<br>A qualitative study | Individual interviews, focus groups, and direct observations<br>A content analysis | Two themes emerged: questions not being challenging and incentive to improve critical thinking in students; incompetency of clinical educators（not prepared and competent for being a role model practical setting）. |
| Günay et al（22）(2018)<br>Turkey | To determine the transfer of theoretical knowledge into clinical practice by nursing students and the difficulties they experience during this process. | 30 nursing students in a university located in the east of Turkey<br>A qualitative research | Focus group interviews<br>The method of content analysis | Three themes emerged: guidance and communication（inadequacy in receiving clinical guidance, lack of appreciation, cooperation); clinical evaluation（expectations changing based on the instructor, injustice in clinical grading）; expectations（to be active in clinical education, love their profession and feel appreciated, to accompany them in the clinical area）. |

**Table 3.** The effect size of themes

| References | Themes | | |
|---|---|---|---|
| | Good teaching literacy | Solid professional competence | Harmonious faculty-student relationship |
| Pearson et al（14） | x | x | |
| Jiang et al（15） | x | x | x |
| Lovrić et al（16） | x | x | x |
| Harms et al（17） | x | x | x |
| Kelly（18） | x | x | x |
| Gustafsson et al（19） | | x | x |
| McSharry et al（20） | x | x | x |
| Yousefy et al（21） | x | x | |
| Günay et al（22） | x | x | x |
| % | 89 | 100 | 78 |

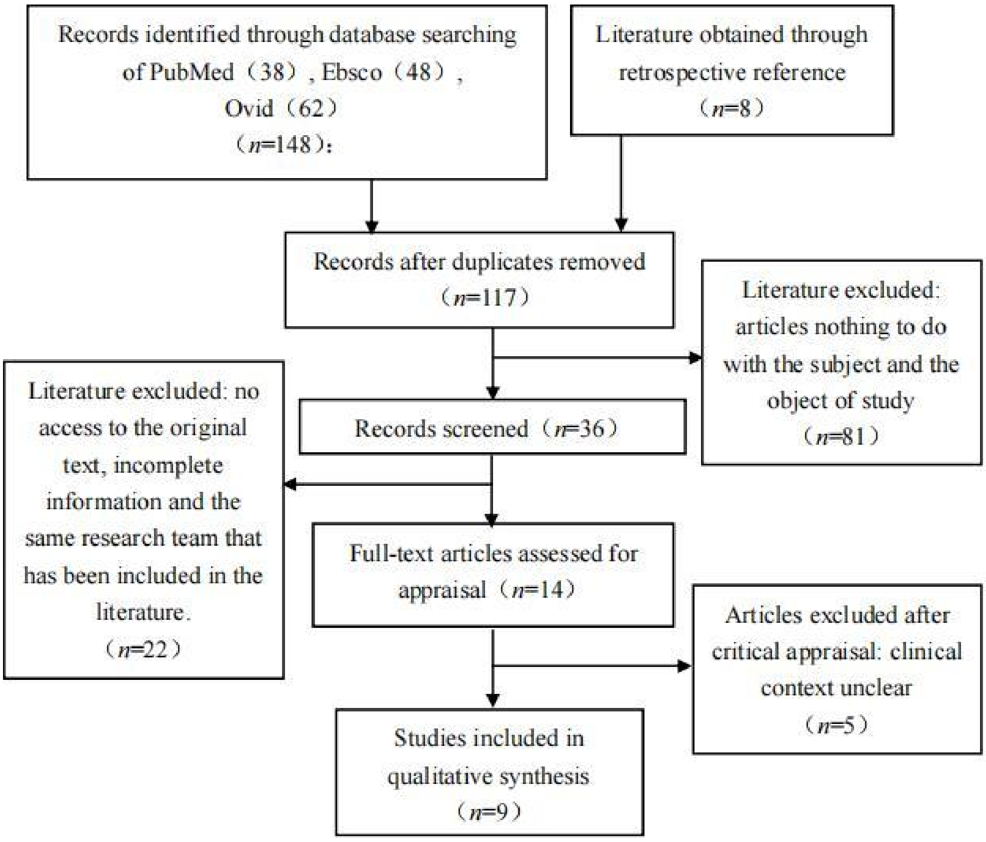


***Fig.1 PRISMA flowchart.***

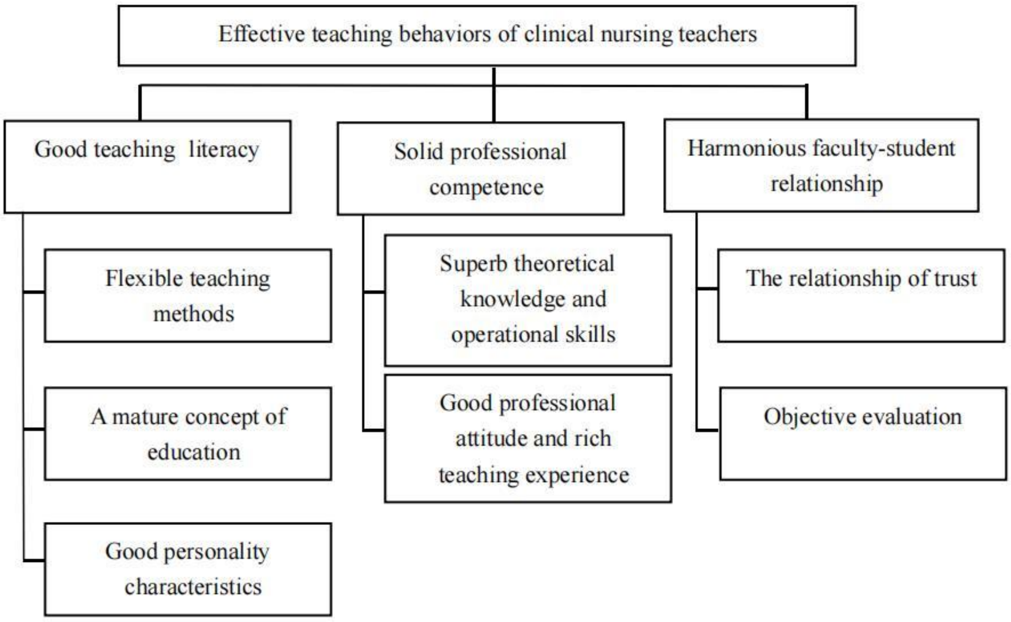


***Fig.2 The relation between meta-synthesis, themes, and categories in the review.***

| Grouping the study findings into categories | Synthesized categories | Themes |
|---|---|---|
| Exploratory conversations<br>Teaching clinical reasoning: preceptors' questions<br>Critical and independent thinking climate | Flexible teaching methods | Good teaching literacy |
| A passion for education<br>Person as pedagogy<br>To be active in clinical education, love their profession and feel appreciated, to accompany them in the clinical area | A mature concept of education | |
| Being empathetic for teaching<br>Positive human qualities<br>Personal characteristics: learner-centered, supportive, engaging, good communicator, respectful, professional<br>Feedback and communication skills: teacher's listening skills, a respectful, calm, co-learner, being straightforward and honest | Good personality characteristics | |
| Possessing professional competence<br>A higher level of teaching ability<br>Supervisors: more than medical experts<br>Clinical teacher knowledge<br>Enabling integration of theory and practice<br>Prepared and competent for being a role model practical setting | Superb theoretical knowledge and operational skills | Solid professional competence |
| A positive learning environment: support for learning, excellence in teaching<br>Continuity: foundation for an effective teaching and learning relationship<br>Talking through practice<br>Guidance and communication: adequacy in receiving clinical guidance, appreciation, cooperation | Good professional attitude and rich teaching experience | |
| Demonstrating the harmonious faculty-student relationship<br>Good interpersonal relations<br>Relationships matter: learner security, a spectrum of admiration<br>Environmental factors: ideal student-teacher ratios, welcoming students and trying to help them out, the importance of peer support<br>Co-operation between placement staff and nurse teacher: being as a member of the nursing team, transmitting his or her pedagogical expertise to the clinical team<br>Relationship between student, mentor and nurse teacher: the common meetings between myself, mentor and nurse teacher being comfortable experience, a climate of the meetings being congenial, focus on the meetings being in my learning needs | The relationship of trust | Harmonious faculty-student relationship |
| Clinical faculties' professional evaluation of student<br>Continuous assessment of the students understanding and performance<br>Clinical evaluation: expectations changing based on the instructor, injustice in clinical grading | Objective evaluation | |

***Fig.3 The meta-aggregative approach from grouping the study findings into categories and synthesizing the categories into themes.***

# 同济大学文件

同济研〔2021〕136号

## 关于公布2021年同济大学研究生教育研究与改革医学专项项目立项名单的通知

各单位：

为深入贯彻立德树人根本任务，落实全国研究生教育工作会议精神，进一步完善我校研究生创新人才培养体系，提高研究生培养质量，按照上海市研究生教育紧缺医学人才培养项目要求，经医学院推荐、专家评审，并报分管校领导批准，现公布2021年同济大学研究生教育研究与改革医学专项项目（34 项）立项名单，其中研究生医学教学案例专项项目 10 项、研究生医学教育管理专项项目16项、研究生医学新建附属医院专项项目4项、研究生医学课程思政专项项目4项。

特此通知

附件：1. 2021年同济大学研究生医学教学案例专项项目一览表

2. 2021年同济大学研究生医学教育管理专项项目一览表

3. 2021年同济大学研究生医学新建附属医院专项项目一览表

4. 2021年同济大学研究生医学课程思政专项项目一览表

同济大学

（电子专用章）

2021年8月14日

附件1

# 2021年同济大学研究生医学教学案例专项项目一览表

| 序号 | 单位 | 项目编号 | 项目名称 | 项目负责人 |
|---|---|---|---|---|
| 1 | 附属第十人民医院 | 2021YXAL01 | 甲状腺结节诊断的“三生三世”(影像-细胞-基因诊断模式) | 徐辉雄 |
| 2 | 附属第十人民医院 | 2021YXAL02 | 基于循证实践理论的一例二尖瓣脱垂患者围手术期护理 | 陈亚梅 |
| 3 | 附属上海市肺科医院 | 2021YXAL03 | 呼吸系统疾病临床护理教学设计与实践 | 毛燕君 |
| 4 | 附属东方医院 | 2021YXAL04 | 老王的2次手术经历 | 张伟英 |
| 5 | 医学院 | 2021YXAL05 | 《心理护理临床实践》教学案例库建设及应用效果研究 | 王艳波 |
| 6 | 附属第十人民医院 | 2021YXAL06 | “前路有光，背后有爱”——社区慢性病患者的健康管理 | 朱晓萍 |
| 7 | 附属第一妇婴保健院 | 2021YXAL07 | 产后出血急救护理高仿真模拟培训案例构建 | 黄蓉 |
| 8 | 附属上海市肺科医院 | 2021YXAL08 | 止血降菌，瓜农重生 | 梁硕 |
| 9 | 附属东方医院 | 2021YXAL09 | 直肠癌--原来你不是痔疮 | 李丹 |
| 10 | 附属第十人民医院 | 2021YXAL10 | 尿源性脓毒血症——与死神争夺的救治 | 张海民 |

附件 2

# 2021年同济大学研究生医学教育管理专项项目一览表

| 序号 | 单位 | 项目编号 | 项目名称 | 项目负责人 |
| --- | --- | --- | --- | --- |
| 1 | 医学院 | 2021YXGL01 | 基于学生发展理论的临床医学“5+3+X”研究生教育质量研究 | 俞徐菊 |
| 2 | 医学院 | 2021YXGL02 | 基于协同创新机制的医学研究生集中选题模式改革的探讨 | 赵一丹 |
| 3 | 附属养志康复医院 | 2021YXGL03 | 康复学科硕士研究生导师遴选标准研究 | 吴恒璟 |
| 4 | 医学院 | 2021YXGL04 | “申请-考核制”背景下专博专培选拔机制的现状、问题及完善路径 | 王玉茹 |
| 5 | 附属上海市肺科医院 | 2021YXGL05 | 医教联合体背景下医师规范化培训的考核评估模式优化研究 | 张育琳 |
| 6 | 附属上海市肺科医院 | 2021YXGL06 | 临床医学专业学位研究生教育管理机制研究 | 杨勐航 |
| 7 | 附属第十人民医院 | 2021YXGL07 | 新时代教改背景下医学院研究生导师考核体系建设研究 | 傅近 |
| 8 | 附属第十人民医院 | 2021YXGL08 | 临床医学专业型博士与专科医师规范化培训并轨制培养模式研究 | 苏斌 |
| 9 | 附属上海市肺科医院 | 2021YXGL09 | 基于问题解决的护理专业学位研究生创新实践能力培养模式的构建 | 李玉梅 |
| 10 | 医学院 | 2021YXGL10 | 学位型博士研究生管理制度的实践与探索 | 李萍 |
| 11 | 附属同济医院 | 2021YXGL11 | 基于 ACGME-I 认证体系的临床培训基地教学质量保障研究 | 黄蕾 |
| 12 | 医学院 | 2021YXGL12 | 护理专业学位研究生核心课程体系建设 | 周薇 |

| | | | | |
|---|---|---|---|---|
| 13 | 附属上海市肺科医院 | 2021YXGL13 | 新媒体学生社区在研究生培养及管理中的作用和评价 | 沈瓅 |
| 14 | 附属杨浦医院 | 2021YXGL14 | 针对新冠疫情需求全科研究生培养研究 | 葛许华 |
| 15 | 附属上海市肺科医院 | 2021YXGL15 | “双一流”大学服务型医学研究生教育管理队伍建设 | 苏春霞 |
| 16 | 附属第十人民医院 | 2021YXGL16 | “三全育人”背景下的医学研究生自我管理平台体系构建 | 郑龙坡 |

附件3

# 2021年同济大学研究生医学新建附属医院专项项目一览表

| 序号 | 附属单位 | 项目编号 | 项目名称 | 项目负责人 |
|---|---|---|---|---|
| 1 | 附属第四人民医院 | 2021YXFS01 | 加强麻醉学科研究生“麻醉与围术期医学并重”理念的新培养模式研究 | 熊利泽 |
| 2 | 附属第四人民医院 | 2021YXFS02 | 以药学服务能力培养为导向的药学专业研究生教育研究和实践 | 娄月芬 |
| 3 | 附属第四人民医院 | 2021YXFS03 | 基于价值医疗的微创外科技术培训体系 | 戚大川 |
| 4 | 附属第四人民医院 | 2021YXFS04 | 利用影像网络教学系统提升研究生与规培生影像理论技能 | 罗禹 |

附件4

# 2021年同济大学研究生医学课程思政专项项目一览表

| 序号 | 附属单位 | 项目编号 | 项目名称 | 项目负责人 |
|---|---|---|---|---|
| 1 | 附属第十人民医院 | 2021YXSZ01 | 基于胜任力的急危重症护理专硕课程思政教育基地建设 | 姜金霞 |
| 2 | 附属同济医院 | 2021YXSZ02 | 生殖医学研究生课程思政建设 | 王炎秋 |
| 3 | 附属东方医院 | 2021YXSZ03 | 融合思政教育的跨专业医疗团队合作培训课程探索 | 俞海萍 |
| 4 | 附属上海市肺科医院 | 2021YXSZ04 | 课堂舞台剧“生命之间”在《肿瘤内科学新进展》课程中的应用 | 苏春霞 |

校长办公室　　　　　　　　　　　　　　　　2021 年 8 月 16 日印发
